# Primary Small Cell Carcinoma of the Esophagus (PSCEC) Associated with Paraneoplastic Sweating Syndrome: A Case Report and Literature Review

**Published:** 2015-11

**Authors:** Seyed Hosein Fattahi Masoum, Noorieh Sharifi, Shirin Taraz Jamshidi, Alireza Sharifian, Reza Rezaee

**Affiliations:** 1 *Department of Thoracic Surgery, Mashad University of Medical Sciences, Iran.*; 2 *Solid Tumor Treatment Research Center, Faculty of Medicine, Mashad University of Medical Sciences, Iran.*; 3 *Department of Anesthesiology, Mashad University of Medical Sciences, Iran.*

**Keywords:** Chemotherapy, Paraneoplastic syndrome, Prognosis, Small cell carcinoma

## Abstract

**Introduction:**

Primary small cell carcinoma of the esophagus (PSCEC) associated with paraneoplastic sweating syndrome is a rare disease characterized with rapid growth rate, metastasis at the time of diagnosis, and poor prognosis. The lung is the most common site for small cell carcinoma but this malignancy includes 0.1% to 1% of all gastrointestinal and 0.8% to 2.7% of esophageal malignancies. So far more than 200 cases of PSCEC have been reported in literature.

**Case Report:**

The patient is a 54-year-old female from the Golestan province who presented with dysphagia, 19 kg-weight loss (from 105 kgs to 86 kgs), and excessive sweating. She was admitted in the thoracic surgery ward, at Ghaem Hospital, in the Mashhad University of Medical Sciences, with a pathological diagnosis of small cell carcinoma. She underwent transhiatal total esophagectomy. Excessive sweating was eradicated after surgery and she was discharged after 13 days without any complication. She received chemotherapy and at her 5-year follow up, she showed no recurrence or metastasis.

**Conclusion:**

PSCEC usually requires chemotherapy with or without surgery. A favorable outcome, with total resection of the lesion combined with chemotherapy, was obtained. However, due to the rarity of the disease there is no definitive choice of treatment.

## Introduction

Primary small cell carcinoma of the esophagus (PSCEC) associated with paraneoplastic syndrome is a rare disease characterized with rapid growth rate, metastasis at the time of diagnosis, and poor prognosis. Clinical manifestations - like other esophageal cancers - include dysphagia (the most frequent manifestation), weakness, weight loss, gastroesophageal reflux, chest pain, and probably also includes the manifestations related to paraneoplastic syndromes.

The incidence of primary small cell carcinoma of the esophagus ranges from 0.1% to 1% of all gastrointestinal tract malignancies and 0.8% to 2.7% of esophageal malignancies (1-4).

The distal two thirds of the esophagus is the most common site of involvement (1,5-10) while the proximal one third is involved in only 5% of patients (11). Patients range in age from 40 to 70 years (our patient is 54-years-old). The prognosis is poor and estimates at a 7 to 19 month survival (1,5,8,11-14). 

In most cases of esophageal cancers, lymph node metastasis is present at the time of diagnosis and in a high proportion of cases there is distant metastasis (30% to 90%). The most common sites for distant metastasis are the liver, peritoneum, and bone (3,12).

## Case Report

The patient is a 54–year-old female from the Golestan province, who demonstrated symptoms of dysphagia and weight loss on July 2008. She suffered from a 19 kg weight loss (from 105 kgs to 86 kgs) and complained of excessive sweating. Physical examination was otherwise normal. The duration of her symptoms was 4 months.

**Fig 1:**
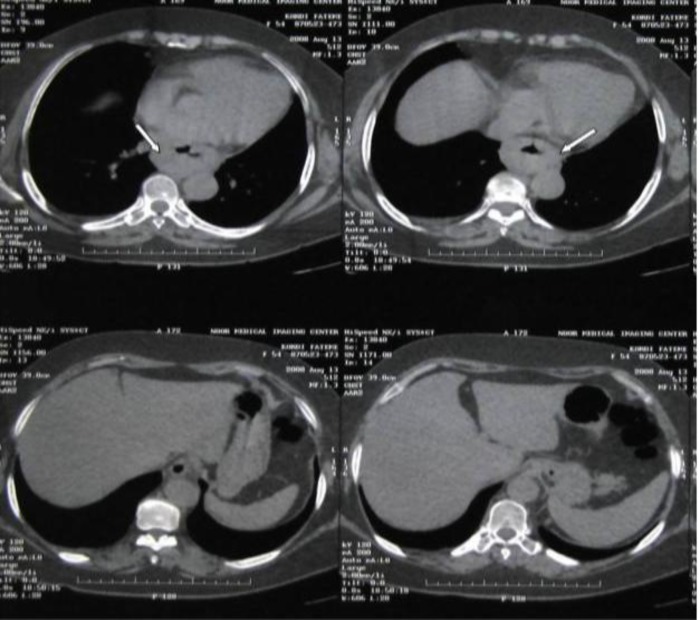
Computed tomography before surgery shows an esophageal mass (arrows

Thyroid function tests and other laboratory data were within normal limits. Computed tomography and esophagoscopy revealed a large vegetative mass extending from 28 to 35 centimeters from her teeth (Fig.1).

**Fig 2:**
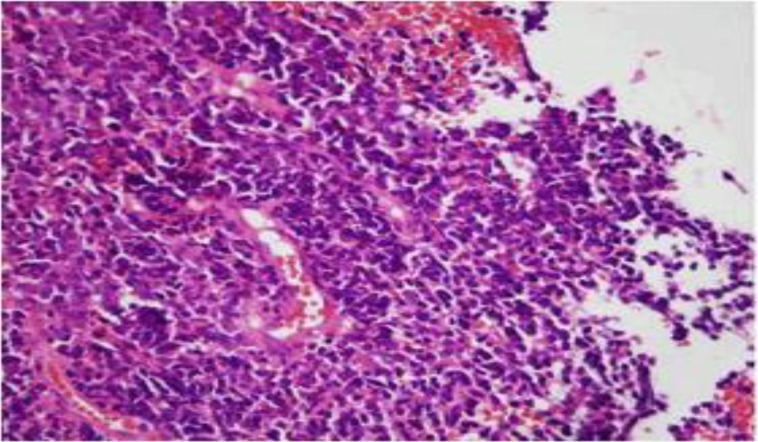
Low magnification of tumor cells with diffuse pattern, small size, and hyperchromatic nuclei; characteristic of small cell carcinoma (H&E stain 100X

**Fig3:**
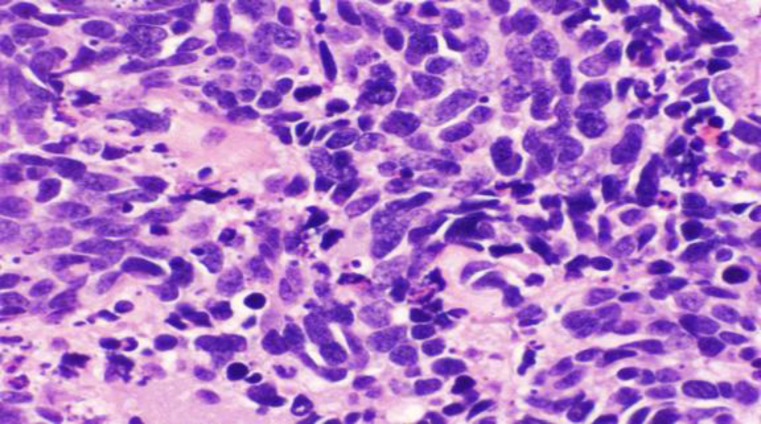
Higher magnification of small cell carcinoma shows oval to spindle cells with hyperchromatic and molded nuclei, scanty cytoplasm, high nucleocytoplasmic ratio, and inconspicuous stroma (H&E stain 400X

A biopsy was performed endoscopically. Histological examination of the biopsy showed small oval to spindle cells with hyperchromatic nuclei, scanty cytoplasm, and high nucleocytoplasmic ratio; in addition to extension to the adventitia and vascular and perineural invasion(Figs 2,3).

Metastasis to one regional lymph node was found. The diagnosis of small cell carcinoma was made by two pathologists and confirmed by immunohistochemistry (IHC). In the IHC study, the tumor cells were immunoreactive for cytokeratin, chromogranin, and synaptophysin, and negative for LCA.

The patient was admitted into the thoracic surgery ward, in Ghaem Hospital, at the Mashhad University of Medical Sciences.

She underwent transhiatal total esopha- gectomy. The severity of her sweating was so high that the linens used during surgery were completely wet. An anesthesiologist performed monitoring during surgery and no complications occurred. Sweating was reduced the day after surgery and was eradicated after 3 days. 7 days later a barium swallow study was performed to ensure the absence of any leakage at the site of anastamosis. The pathologic stage of the tumor was T2N1Mx according to AJCC TNM classification. The patient was discharged 13 days later and underwent a complete course of chemotherapy.6 chemotherapy courses were administered consisting of etoposide 100 mg/m^3^/3 days and cisplatin 80-100 mg/m^3^. Her 5 year follow up revealed no complications, local recurrance, or distant metastasis. The patient now lives a normal life.

## Discussion

Primary small cell carcinoma of the esophagus associated with paraneoplastic syndrome presents like other esophageal malignancies and the diagnosis is based on histolagical examinations (7). 

Morphological appearance and biological behavior of this tumor resemble the more common small cell carcinoma of lung (3). 

Small cell carcinoma of the esophagus was first reported in 1952 by Mckeown (4). This tumor is rare and includes 0.4% to 2.7% of all esophageal carcinoma. In gross examination it usually presents as a polypoid

mass with an ulcerated surface. Clinical manifestations are similar to other esophageal tumors and there are few reports of paraneoplastic syndromes with ectopic hormone secretion (3,8). Paraneoplastic syndromes are seen in patients with undiagnosed cancers and are usually eliminated after diagnosis and treatment. There is an increased risk for paraneoplastic syndromes in patients with a family history of breast and colon cancer. Nonspecific syndromes can change clinical features of the tumor and are considered to be unfavorable prognostic factors.

Itching is the most common cutaneous presentation of paraneoplastic syndromes. Shingles ichthyiosis, flushes, alopecia, and hyperthricosis are also reported.Acanthosis nigricans and multiple seborrheic keratosis are specifically present in metastatic malignant melanomas and visceral tumors such as malignancies of the pancreas (3,9,15).

So far excessive sweating as a paraneoplastic syndrome of PSCEC has not been reported.

Sun and colleagues reported 73 cases of PSCEC that underwent surgery and chemotherapy for stages I and II and chemotherapy in addition to radiation for stages III and IV. 65 of 73 cases underwent radical surgery. The 1, 3, and 5 year survival rates were 50.7%, 13.7%, and 8.2% respectively (10).

Treatment protocol is controversial due to the rarity of the disease and inadequate data; however, the general agreement is that surgical resection and radiation therapy provide a good short-term outcome. Long-term prognosis is still unfavorable.

Situ et al reported 44 cases and recommended radical esophagectomy and extensive lymphadenectomy in patients with local PSCEC (16). In Mcfadden’s study, the average survival rate was 8 months in 29 patients that underwent surgery (11). Nemato et al have reported a 5 month survival rate for 20 PSCEC cases that received radiation therapy (12). Casas et al have reported 199 PSCECs with an 8-month survival rate (2).

Chemotherapy after surgery has an important role in combination therapy of PSCEC. In Sun’s study, 4 patients with postsurgical chemotherapy survived for more than 10 years (10). Beyer et al reported a 5.3 month survival rate (5). In recent studies there are considerable differences in 1, 3, and 5 year survival rates in patients suffering from stage I/II cancers compared with stage III/IV cancers (10).

In Russel Gollard’s study, a favorable response has been obtained with neoadjuvant chemotherapy using cisplatin and etoposide in 2 patients with limited stage PSCEC although they only survived for a short time. In general, for limited stage diseases, survival time is less than one year and few cases survive for more than 2 years (17).

Chemotherapy combined with radiation therapy, without debilitating surgery may provide long-term survival. 

With the exception of a few cases with long-term survival, most patients survived less than 1 year and surgery is was recommended due to the lack of effective agents to control both local and advanced disease. Chemotherapy combined with radiation therapy is the choice (17).

In Jing Ping Yun’s study from china, PSCEC contributes to 0.5% of esophageal malignancies. In 21 cases they combined surgery, chemotherapy, and radiation therapy and observed a mean survival rate of 18 months (from 3 to 71 months). 28.6% survived for more than 2 years (13). Medgyesy et al considered surgery combined with multimodality adjuvant therapy as a curative treatment (8). So far, few patients have completely responded and survived for a long time. Takuya Shimoda et al performed chemotherapy and radiation therapy (full dose 60GY) and their patients remained disease free for 6 years (9). 

## Conclusion

The most important prognostic factor in patients with PSCEC with extensive involvement is the tolerance of local and/or systemic treatments. Combined chemotherapy and radiation therapy in addition to surgery have been used for limited disease or chemotherapy resistant cases.

In our experience, we recommend surgery in all esophageal malignancies including PSCEC and also adjuvant therapy (chemotherapy). This was performed in this case and the patient is now symptom free for more than 5 years.
